# Performance Characteristics of Combinations of Host Biomarkers to Identify Women with Occult Placental Malaria: A Case-Control Study from Malawi

**DOI:** 10.1371/journal.pone.0028540

**Published:** 2011-12-12

**Authors:** Andrea L. Conroy, W. Conrad Liles, Malcolm E. Molyneux, Stephen J. Rogerson, Kevin C. Kain

**Affiliations:** 1 Sandra A. Rotman Laboratories, McLaughlin-Rotman Centre for Global Health, University Health Network-Toronto General Hospital, University of Toronto, Toronto, Ontario, Canada; 2 Malawi-Liverpool-Wellcome Trust Clinical Research Programme, Blantyre, Malawi; 3 College of Medicine, University of Malawi, Blantyre, Malawi; 4 School of Tropical Medicine, University of Liverpool, Liverpool, United Kingdom; 5 Department of Medicine, University of Melbourne, Royal Melbourne Hospital, Parkville, Victoria, Australia; 6 Tropical Disease Unit, Division of Infectious Diseases, Department of Medicine, University of Toronto, Toronto, Ontario, Canada; Lile 2 University, France

## Abstract

**Background:**

Because of its propensity to sequester in the placental intervillous space, *Plasmodium falciparum* can evade detection by peripheral smear in women with placental malaria (PM). We evaluated host biomarkers as potential indicators of occult PM infections.

**Methods and Findings:**

Using a case-control design, we evaluated the ability of biomarkers to identify PM in the absence of circulating peripheral parasites (n = 24) compared to placental smear-negative controls (n = 326). We measured levels of biomarkers (C3a, C5a, CRP, angiopoietin-1, angiopoietin-2, sTie-2, sEndoglin, VEGF, sFlt-1, tissue factor, and leptin) in maternal peripheral plasma at delivery. Using ROC curve analysis, we assessed the ability of clinical parameters and biomarkers to accurately detect PM infections identified by placental smear. We show that decreases in sFlt-1 and leptin and increases in CRP were associated with occult PM infections (p<0.01) and correlated with placental parasitaemia (p<0.01). Individually, all markers had moderate ability to diagnose occult PM infections with areas under the ROC between 0.62 and 0.72. In order to improve diagnostic performance, we generated simple scoring systems to identify PM infections using either a clinical score (0–2), a biomarker score (0–3) or a clinical plus biomarker score (0–5). The combinatorial model that incorporated both clinical parameters and biomarkers had an area under curve (AUC) of 0.85 (95% CI, 0.81-0.89), which was significantly better at identifying occult PM infections than the clinical score alone (p = 0.001).

**Conclusion:**

These data suggest that host biomarkers in the maternal peripheral blood may improve the detection of PM in the absence of peripheral parasitaemia.

## Introduction

Every year 85 million pregnant women are at risk of infection by the malaria parasite *Plasmodium falciparum* [1]. Malaria in pregnancy (MIP) may lead to adverse consequences for both the mother and the fetus, including severe maternal anemia, spontaneous abortion, stillbirth and low birth weight (LBW). In areas of unstable transmission of *P. falciparum*, mothers are at increased risk of severe malarial disease, including cerebral malaria and hypoglycaemia [2]. As the level of endemicity and prior clinical immunity to malaria increases, MIP is more likely to be asymptomatic or paucisymptomatic [2].

The proclivity of *P. falciparum*-infected erythrocytes (IE) to bind to chondroitin-sulfate A (CSA) in the placental intervillous space [3] can make it difficult to detect placental malaria (PM) by either microscopic examination of Giemsa-stained peripheral blood smears or point-of-care rapid diagnostic testing (RDT) alone [4], [5], [6]. Occult placental malaria may still lead to adverse pregnancy outcomes. One strategy to decrease pregnancy-related malaria complications is intermittent preventive treatment in pregnancy (IPTp), the widespread administration of antimalarials (typically sulfadoxine-pyrimethamine) to pregnant women irrespective of their infection status at two or more scheduled antenatal visits during pregnancy [7]. Although IPTp has reduced infant low birth weight by 43% [2], declining malaria transmission and increasing resistance to sulfadoxine-pyrimethamine will change the cost-benefit ratio for IPTp, potentially favoring intermittent screening and treatment (IST) involving newer, more costly and potentially less safe therapeutic agents [8]. Given the limitations of traditional and field diagnostics to detect placental malaria, the identification of biomarkers present in the maternal peripheral blood that could identify PM in the absence of patent peripheral parasitemia, could minimize unnecessary drug treatment during pregnancy, improve the sensitivity of IST to detect placental malaria, reduce drug pressure and the selection of resistant parasites, and improve maternal and fetal outcomes.

Because occult PM induces a local host response in the placenta, and soluble components of the placental compartment may circulate in the peripheral blood, we evaluated a number of host proteins as potential candidate biomarkers for the detection of placental malaria, in the absence of detectable circulating parasites. We measured markers of inflammation (C-reactive protein), complement activation (C3a, C5a), angiogenesis (angiopoietin-1, angiopoietin-2, soluble Tie-2, vascular endothelial growth factor (VEGF), soluble fms-like tyrosine kinase-1 (sFlt-1), and soluble Endoglin), coagulation (tissue factor), and nutrient availability (leptin). Our results indicate that decreases in sFlt-1 and leptin and an increase in CRP in peripheral plasma were associated with the presence of placental parasites.

## Methods

### Ethics Statement

Ethical approval for this study was granted from The College of Medicine Research Ethics Committee in Blantyre, Malawi (COMREC) and all women gave written informed consent for enrolment into the study.

### Study Population

From 2001-2006, a cross-sectional study was carried out in Blantyre, Malawi, in which pregnant women were prospectively recruited at the Gogo Chatinkha Banda Maternity Unit of Queen Elizabeth Central Hospital. Upon delivery, a thick blood film was prepared from the cut surface of the placenta, and women were recruited as cases if they were positive for placental malaria, delivered a live singleton newborn, and had a peripheral blood thick film that was negative for malaria parasites. Twenty-one women were excluded from the study because they had malaria parasites detected by peripheral smear, but not placental smear. Age and gravidity-matched controls (peripheral and placental smear negative for malaria) were chosen in a 2∶1 ratio. Controls enrolled based on a negative placental smear were not reclassified as cases based on the identification of parasites by subsequent histology, as these sub-microscopic infections were not associated with significantly different clinical outcomes than histology negative controls in this cohort. Placental and peripheral EDTA plasma samples were collected after women had given their informed consent, and samples were stored at -80°C until testing.

### Biomarker ELISAs

Plasma concentrations of biomarkers C5a, angiopoietin (Ang)-1, Ang-2, sTie-2, sEndoglin, VEGF, sFlt-1, leptin, and CRP (DuoSets, R&D Systems, Minneapolis, MN) were measured in peripheral plasma by ELISA, as described [9]. C3a (capture: clone 354113, detection: purified PAb, Goat IgG) and tissue factor (capture: clone 323514, detection: PAb, goat IgG) were measured using antibody pairs (R&D Systems, Minneapolis, MN) with biotin swine anti-goat IgG (human and mouse adsorbed, clone CLCC50015) as a secondary antibody as described previously [9]. ELISAs were performed in 2009 by a single experienced technician who was blinded to the microscopy results during sample testing.

### Statistical Analysis

GraphPad Prism v5, SPSS v16, and MedCalc software were used for analysis. Comparisons of continuous variables were performed using the Mann-Whitney U test and Spearman rank correlation coefficient. Comparisons of proportions were performed using Pearson Chi-square test or Fisher's exact test, as appropriate. Odds ratios (OR) were calculated using Pearson's Chi-square. The diagnostic accuracy of biomarkers and laboratory findings were assessed using non-parametric receiver operating characteristic (ROC) curves, and the areas under the ROC (AUC) were compared using the method of Delong et al. [10]. Biomarker cut-points were determined using the Youden index (J  =  max[sensitivity + specificity – 1]).

## Results

### Description of Study Population

A total of 465 women with live singleton deliveries were included in the study. Of these women, 326 were peripheral and placental smear-negative for malaria and 139 were PM-positive based on placental smears. Of the 139 placental smear-positive women, 24 had negative peripheral smears. Over half (53.4%) of the placental smear-negative women had histologic evidence of active malaria infection (parasites ± pigmented fibrin/mononuclear cells) and 16% of women had histologic findings indicating past infection [11]. However, there were no differences in peripheral blood biomarkers, maternal hemoglobin, or fetal outcome (gestational age or birth weight) in placental smear-negative women with histologically defined PM infections compared to those with no detectable placental parasites (Supplemental information, **Table S1**). Tissue factor was reduced in histology-positive PM compared to histology-negative controls; however, this result was not consistent in women with smear positive infections and the biomarker had poor discriminatory ability. Histology-only infections were not associated with clinical signs of disease or clinically relevant outcomes (e.g. low birth weight) in this population. Therefore, women were classified according to the study design and cases represent smear positive infections. In order to evaluate biomarkers in maternal peripheral plasma that were the most sensitive for placental-specific infections, the 326 peripheral and placental smear-negative women were compared to women who were PM positive without detectable infections by peripheral smear. The study population is described in **Table 1**.

**Table 1:** Description of Study Population.

	Placental smear positive PMN = 24	Placental and peripheral smear negativeN = 326	P-value
**Age (years)**	20.5 (18.3–25.3)	20.0 (18.0–23.0)	0.603
**Gravidity**	1 (1–3.75)	1 (1–2)	0.580
**% malaria-positive by histology**	100	53.4	<0.0001
**Total # antimalarial doses** †	2.0 (2.0–2.25)	2 (1–3)	0.575
**Weight (kg)**	56.0 (50.25–60.25)	56.0 (51.0–60.0)	0.754
**Height (cm)**	155 (150–159)	154 (150–158)	0.743
**Temperature (°C)**	36.5 (36.0–37.0)	36.1 (36.0–36.6)	0.076
**Hemoglobin (g/dL)**	10.9 (10.1–12.2)	12.3 (11.1–13.5)	0.003
**Any febrile symptoms in last 7 d, n (%)**	15 (62.5)	63 (19.3)	<0.0001
Fever in last 7 d, n (%)	9 (37.5)	15 (4.6)	<0.0001
Chills in last 7 d, n (%)	9 (37.5)	22 (6.7)	<0.0001
Headache in last 7 d, n (%)	15 (62.5)	56 (17.2)	<0.0001
**Weight of baby (kg)**	2.9 (2.5–3.1)	3.0 (2.7–3.3)	0.036
**Weeks gestation at delivery**	38 (36–40)	39 (38–40)	0.030
**Mononuclear cell count** *	18.5 (13.25–30.75)	3 (0–13)	<0.0001
**Mononuclear cell pigment score (0–4)** *	1.0 (0.25–2.0)	0 (0–0)	<0.0001
**Fibrin pigment score (0–4)** *	2.0 (0–3.0)	0 (0–1)	<0.0001

Data are presented as median (interquartile range) unless otherwise indicated.

Groups were compared using the Mann-Whitney U test (for continuous variables) or Pearson Chi-square (for nominal variables).

† Number of doses of SP recorded on antenatal clinic card.

*Assessed using a semi-quantitative scale by a single observer.

Women with placental smear-positive PM had a significantly lower median peripheral-blood hemoglobin concentration ([Hb]) than placental smear-negative women (10.9 g/dL vs. 12.3 g/dL, p = 0.003, Mann-Whitney U test) (**Table 1**). Anemia (peripheral blood [Hb] <11 g/dL) in the mother was associated with a 3.3-fold (95% CI, 1.4–7.6) increased odds of PM. Women with PM more commonly reported non-specific symptoms (fever, chills, headache) within one week prior to delivery (p<0.0001) (**Table 1**). The presence of one or more of the above symptoms was associated with a 7.0-fold (2.9–16.6) increased odds of placental infection (p<0.0001).

### sFlt-1, Leptin and CRP are Clinically Informative Biomarkers of Placental Malaria Infection

**Figure 1:**
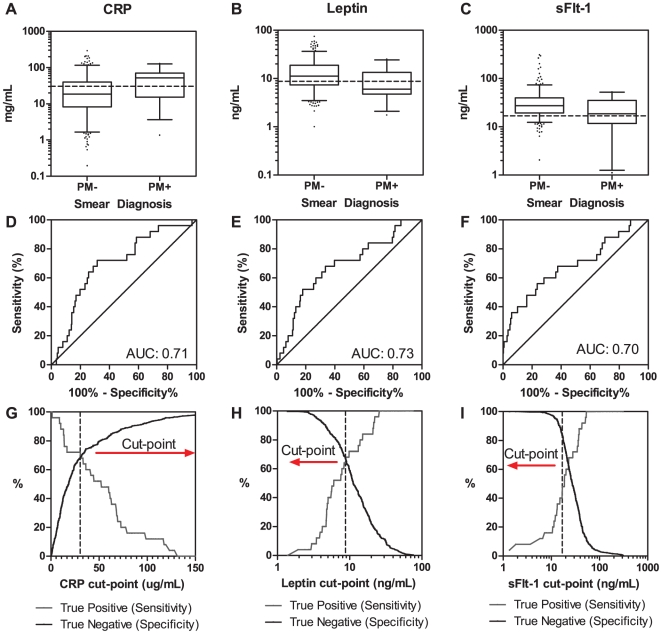
Biomarkers of Occult Placental Malaria Infection. (A-C) Box plots showing the median (IQR) of peripheral blood biomarkers (whiskers denote the 5-95% percentiles and outliers are plotted as dots), with the associated (D-F) receiver operating characteristic (ROC) curves and (G-I) decision plots of sensitivity and specificity generated from the ROC curves. Diagnostic accuracy was assessed using receiver operating characteristic (ROC) curves and determining the area under the ROC curve (AUC). The AUC were compared and all three biomarkers had comparable diagnostic performance (method of Delong et al.). The dotted lines represent the cut-point for dichotomizing the biomarkers, as defined using the Youden index.

We included a number of biomarkers in our analysis that have previously been shown to be altered in the peripheral blood in women with placental malaria, including CRP [6], [12], complement C5a [13], the angiopoietin (Ang) family of Ang-1, Ang-2 and a soluble form of their cognate receptor, Tie-2 (sTie-2) [14], anti-angiogenic proteins sFlt-1 and sEndoglin [15], and leptin [16]. Median Ang-1, sFlt-1, and leptin levels were significantly lower, and median CRP levels were significantly higher in women with placental malaria than in uninfected controls (**Figure 1** **,** **Table 2**). These markers were significantly correlated with quantitative placental parasite density on placental blood smear (Spearman's rho, p-value): Ang-1, -0.110, p = 0.044; sFlt-1, -0.174, p = 0.001; leptin, -0.203, p<0.001; and CRP, 0.188, p<0.001. Ang-2 was the only marker significantly associated with placental mononuclear cell counts, as assessed by histology (0.141, p = 0.009).

**Table 2:** Peripheral Plasma Biomarkers in Occult Placental Malaria Infections.

	Infected	Uninfected	P-value	AUC^a^	Cut-point	Sensitivity	Specificity
**CRP** b	60.2 (16.7–72.7)	18.5 (8.2–39.7)	0.002*	0.71 (0.66–0.76)	30.5	73.9 (51.6–89.8)	68.3 (62.9–73.4)
**C3a** b	2.0 (1.6–4.4)	2.4 (1.6–4.5)	0.704	0.56 (0.50–0.60)	6.4	50.0 (29.1–70.9)	66.0 (60.5–71.1)
**C5a**	63.0 (32.2–76.1)	58.1 (39.9–78.4)	0.307	0.53 (0.47–0.58)	35.4	34.8 (16.4–57.3)	81.5 (76.8–85.6)
**Ang-1**	13.7 (6.7–20.6)	17.9 (9.4–32.2)	0.037	0.62 (0.57–0.67)	8.5	43.5 (23.2–65.5)	79.9 (75.1–84.2)
**Ang-2**	4.4 (2.8–7.7)	5.1 (3.2–7.9)	0.555	0.54 (0.49–0.59)	2.9	34.8 (16.4–57.3)	84.3 (79.9–88.1)
**sTie-2**	28.9 (22.1–32.1)	24.6 (20.0–30.4)	0.311	0.60 (0.55–0.65)	27.8	60.9 (38.5–80.3)	64.6 (59.1–69.8)
**sEndoglin**	43.0 (29.0–72.6)	46.7 (36.5–57.5)	0.819	0.49 (0.43–0.54)	69.95	30.4 (13.2–52.9)	90.6 (86.8–93.6)
**sFlt-1**	16.9 (11.4–34.7)	27.3 (19.3–39.7)	0.003*	0.70 (0.64–0.74)	16.9	52.2 (30.6–73.2)	83.7 (79l2–87.6)
**VEGF**	0.10 (0.04–0.23)	0.14 (0.06–0.41)	0.242	0.57 (0.52–0.63)	0.075	47.8 (26.8–69.4)	70.2 (64.9–75.2)
**Tissue Factor**	0.10 (0.06–0.14)	0.08 (0.05–0.14)	0.370	0.56 (0.50–0.61)	0.043	95.5 (77.2–99.9)	23.8 (19.1–29.4)
**Leptin**	5.6 (4.7–9.8)	11.2 (7.4–18.9)	<0.001*	0.73 (0.67–0.77)	8.8	73.9 (51.6–89.8)	66.5 (61.0–71.6)

Groups (infected vs. uninfected) were compared using the Mann-Whitney U test.

*Significant (p<0.05) after Holm's correction for 11 pair-wise comparisons. AUC- area under the receiver operating characteristic curve (95% CI),

a Non-parametric estimation (DeLong et al., 1988 estimated using binomial exact method (MedCalc)). Biomarkers are ng/mL unless otherwise indicated-

b mg/mL.

We assessed the diagnostic accuracy of our biomarkers using receiver operating characteristic (ROC) curve analysis (**Figure 1**). sFlt-1, leptin and CRP all had AUC ≥ 0.70 for discrimination between cases and controls, and provided significant discrimination in univariable analysis after applying a correction factor for multiple comparisons (**Table 2** **,** **Figure1**). We used the Youden index to create a cut-point for each biomarker, and low sFlt-1 (<16.9 ng/mL), low leptin (<8.8 ng/mL) and high CRP (>30.5 mg/mL) were associated with increased odds ratios (95% CI) of placental malaria of 4.7 (2.0–11.2), 4.6 (1.8–11.5), and 6.1 (2.3–16.0) respectively. The sensitivity and specificity for the biomarkers at their respective cut-points are shown in **Table 2**.

### Predicting Placental Malaria using Combinations of Biomarkers

We hypothesized that combinations of biomarkers would improve diagnostic accuracy, as each protein could contribute unique information to a predictive model. Therefore, we generated a biomarker score based on dichotomization and summation of the individual biomarkers with the best performance: sFlt-1, leptin, and CRP. For each marker, one point was assigned if the value was lower (sFlt-1, leptin) or higher (CRP) than the corresponding cut-point, and zero points were assigned otherwise. None of the dichotomized biomarkers were significantly correlated (p>0.05 for all comparisons using Spearman correlation).

**Figure 2:**
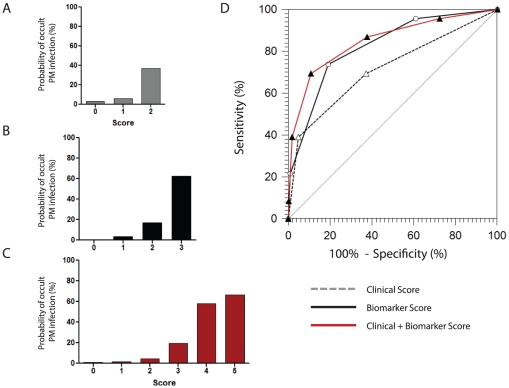
Combinations of Biomarkers and Clinical Parameters Improve Diagnostic Accuracy. (A–C) We generated a simple scoring system based on the summation of dichotomous variables that were significantly associated with the presence of smear positive parasites in the placenta, but not the periphery. (A) The clinical score (0–2) consisted of maternal anemia (1) and non-specific febrile symptoms (any one of: fever, chills, headache = 1). (B) The biomarker score (0–3) consisted of high CRP levels (>30.5 mg/mL = 1), low leptin levels (<8.8 ng/mL = 1), and low sFlt-1 levels (<16.9 ng/mL = 1). (C) The clinical score and biomarker score were integrated to generate the final score (0–5). (D) The diagnostic ability of the different scoring systems was assessed using ROC curve analysis. The clinical + biomarker score had an AUC of 0.85 (95% CI, 0.81-0.89) and was significantly better than the clinical score at discriminating between women with occult PM infections compared to smear-negative controls, p = 0.001 (method of Delong, et al.).

ROC curve analysis of the 3 biomarker score yielded an AUC (95% CI) of 0.83 (0.78-0.86). A score of 1 or more was able to identify 95.7% of all infections (sensitivity), and a score of 3 had a specificity of 100% (**Figure 2**). A score of 2 had a sensitivity and specificity of 73.9% and 80.9%, respectively. Evaluation of more parsimonious biomarker scoring systems showed that 2-biomarker scores incorporating leptin and CRP (AUC = 0.78 [0.73–0.82]), as well as sFlt-1 and CRP (AUC = 0.78 [0.74–0.83]), provided similar discriminative ability to the 3-biomarker score.

Next, we sought to integrate our biomarkers into models that included established clinical parameters associated with placental infection, including maternal anemia and febrile symptoms. To do this, we first generated a clinical score (0–2) based on the presence of maternal anemia and non-specific febrile symptoms. The clinical score had an AUC of 0.72 (0.67–0.77) (**Figure 2**). Then by combining the 3-marker biomarker score with the clinical score (into a five-point score), we were able to achieve an AUC of 0.85 (0.81–0.89), which was significantly better at identifying PM infections than the clinical score alone (p = 0.001) (**Figure 2**), but not better than the 3-marker biomarker score (p = 0.39).

## Discussion

It is well-established that malaria in pregnancy increases the risk of low birth-weight delivery. In some women with placental malaria, parasites cannot be detected in the peripheral blood. Such infections are commonly asymptomatic or paucisymptomatic, yet they are still associated with adverse effects on pregnancy outcome. The existence of such ‘hidden’ *P. falciparum* infections makes it difficult to assess the true impact of malaria in pregnancy and poses challenges to the implementation of proposed programs of intermittent screening and treatment [17].

In this study we assessed the utility of host biomarkers to identify occult PM infections in a case-control study of Malawian women. We compared peripheral plasma levels of these biomarkers between women with placental smear-positive *P. falciparum* infections (peripheral blood smear negative) and placental smear-negative controls. We identified three biomarkers that were significantly associated with the presence of placental parasites (sFlt-1, leptin, CRP) and evaluated the diagnostic performance of these biomarkers alone and in combination using a simple scoring system. The biomarker score was associated with occult PM infections, had good diagnostic performance, and allowed more accurate prediction of placental malaria than a score based on clinical criteria alone. Our data suggest that a combinatorial biomarker score could be used to identify women likely to benefit from targeted anti-malarials during pregnancy.

sFlt-1 is an anti-angiogenic protein that is an alternatively spliced variant of Flt-1 (VEGFR-1). Elevated sFlt-1 has been implicated in the pathophysiology of placental malaria [13], [15], [18] and has also been identified as a biomarker that is elevated in pregnancy-associated hypertension and preeclampsia [15], [19], [20], [21]. We observed a substantial decrease in peripheral sFlt-1 levels in PM infections that were peripheral smear negative compared to controls. The identification of decreased sFlt-1 in maternal peripheral blood in malaria is a novel finding, and these data suggest that sFlt-1 may be particularly useful as a blood test in pregnancy in malaria-endemic areas, with decreases in sFlt-1 being associated with occult PM infections and elevations in sFlt-1 predicting the onset of preeclampsia. Further studies will be required to assess the utility of this marker in populations where PM and preeclampsia may interact to drive adverse pregnancy outcomes [15].

Leptin is a peptide hormone that influences energy homeostasis and regulates neuroendocrine function, in addition to its role as an inflammatory molecule in immune function. The syncytiotrophoblast produces an abundance of leptin in later stages of pregnancy and decreased leptin levels in pregnancy have been associated with fetal growth restriction [22], [23]. We observed a significant reduction in maternal leptin levels in occult PM compared to controls. As most placentally-derived leptin is released into the maternal circulation [24], we postulate that PM infection may reduce the release of leptin into the maternal circulation.

CRP is an acute phase reactant and a non-specific marker of inflammation. Our results indicate that CRP is elevated in occult PM infections, which is consistent with a previous report from Gabon that described elevated CRP in PM, but not sub-microscopic infections (assessed by PCR) [25]. In this study, CRP was the best single predictor with the highest AUC. It is an attractive candidate biomarker given its commercial availability and widespread use in other conditions. However, owing to its lack of specificity, the utility of CRP as a biomarker of occult PM requires further investigation in studies that include other common infections in pregnancy such as chorioamnionitis [26].

Combinations of biomarkers have sometimes been shown to provide better diagnostic or prognostic accuracy than single markers, particularly if drawn from distinct pathobiological pathways [27]. In this study, we combined markers of angiogenesis (sFlt-1), metabolism (leptin), and inflammation (CRP) to improve identification of occult PM infections. By combining the three biomarkers with the highest individual AUC, we were able to achieve better predictive ability than with any of the biomarkers used on their own (comparison of AUC between 3 biomarker model and individual models by method of Delong et al., p<0.05). Assays for both CRP and sFlt-1 have been commercialized and are available for immediate use. We were able to integrate the biomarker data with easily obtained clinical data (self-reported febrile symptoms, anemia) to generate a simple scoring system with an AUC of 0.85.

While the results of this study are promising, they require validation in other populations with differing levels of malaria endemicity. In this population, submicroscopic PM infections were not associated with differences in biomarkers or with maternal (anemia, signs and symptoms of disease) or fetal outcome (birth weight); however, this may not be the case in all populations. The biomarkers that we have examined will need to be assessed prospectively together with other inflammatory biomarkers that have recently been associated with placental malaria [28], and alongside malaria rapid diagnostic tests and PCR to compare performance and to determine whether they could be combined to improve PM diagnosis. The biomarkers discussed in this study will need to be evaluated at earlier stages in pregnancy, at times when presumptive therapy might usefully be applied, as well as in the context of other clinically important infections, in particular HIV.

The case-control design of this study enabled us to look at biomarkers in a broader context by removing the effect of age and gravidity on susceptibility to PM. Future studies should validate these biomarkers in a prospective study.

### Conclusion

Biomarkers circulating in the peripheral blood of pregnant women may provide more accurate prediction of placental malaria than clinical indicators alone, in women without peripheral parasitaemia. Analysis of biomarkers in peripheral blood could contribute to the accurate identification of women most in need of antimalarial therapy in pregnancy, and thereby facilitate programs of intermittent screening and therapy aimed at reducing the impact of malaria on the pregnant woman and unborn child.

## Supporting Information

Table S1 **Clinical and biomarker parameters in histologically defined PM.**
(DOC)
