# Supplementary material for: Performance Characteristics of Combinations of Host Biomarkers to Identify Women with Occult Placental Malaria: A Case-Control Study from Malawi
Source: PLoS One. 2011 Dec 12;6(12):e28540. doi: 10.1371/journal.pone.0028540 (PMC3236186; doi:10.1371/journal.pone.0028540)
Supplement: Table S1 — Clinical and biomarker parameters in histologically defined PM. (DOC) [file pone.0028540.s001.doc]

**Table S1. Clinical and biomarker parameters in histologically defined PM**

|  | **Histology Positive**  **N=198** | **Histology Negative**  **N=152** | **P-value*** |
| --- | --- | --- | --- |
| **Clinical Parameters** |  |  |  |
| Temperature (^o^C) | 36.2 (36.0-36.6) | 36.1 (36.0-36.6) | 0.505 |
| Hemoglobin (g/dL) | 12.4 (10.9-13.6) | 12.0 (10.9-13.3) | 0.258 |
| Febrile symptoms in last 7 d, n (%) | 44 (22.2) | 34 (22.4) | 0.974 |
| Weight of baby (kg) | 3.0 (2.7-3.3) | 3.0 (2.7-3.3) | 0.989 |
| Weeks gestation at delivery | 39.0 (38.0-40.0) | 38.0 (38.0-40.0) | 0.179 |
| **Biomarker Measurements** |  |  |  |
| CRP ^a^ | 20.4 (10.1-51.6) | 16.9 (7.3-44.1) | 0.305 |
| C3a ^a^ | 2.2 (1.6-4.4) | 2.5 (1.6-4.7) | 0.385 |
| C5a | 56.0 (38.5-72.1) | 62.8 (40.0-81.1) | 0.044 |
| Ang-1 | 17.2 (9.2-8.6) | 18.9 (9.3-31.1) | 0.546 |
| Ang-2 | 5.3 (3.3-8.6) | 4.6 (3.2-7.3) | 0.231 |
| sTie-2 | 24.0 (19.9-29.9) | 25.6 (20.8-31.5) | 0.070 |
| sEndoglin | 46.6 (36.4-57.5) | 46.8 (35.5-57.9) | 0.919 |
| sFlt-1 | 27.6 (18.9-39.8) | 25.6 (18.6-38.8) | 0.460 |
| VEGF | 0.13 (0.04-0.35) | 0.16 (0.07-0.53) | 0.075 |
| Tissue Factor | 0.08 (0.04-0.12) | 0.09 (0.06-0.17) | 0.002* |
| Leptin | 10.8 (6.7-18.7) | 11.1 (7.7-18.7) | 0.674 |

Data are presented as median (interquartile range) unless otherwise indicated.

Groups were compared using the Mann-Whitney U test (for continuous variables) or Pearson Chi-square (for nominal variables). Biomarkers are ng/mL unless otherwise indicated-^a^ mg/mL

* p<0.05 following Holm’s correction for multiple comparisons
